# Autophagy regulates long‐term cross‐presentation by murine dendritic cells

**DOI:** 10.1002/eji.202048961

**Published:** 2021-02-10

**Authors:** Nataschja I. Ho, Marcel G. M. Camps, Martijn Verdoes, Christian Münz, Ferry Ossendorp

**Affiliations:** ^1^ Department of Immunology Leiden University Medical Center Leiden The Netherlands; ^2^ Department of Tumor Immunology Radboud Institute for Molecular Life Sciences Radboud University Medical Center Nijmegen The Netherlands; ^3^ Viral Immunobiology, Institute of Experimental Immunology University of Zürich Zürich Switzerland

**Keywords:** autophagy, cross‐presentation, DC, LC3, MHCI

## Abstract

Autophagy has been reported to be involved in supporting antigen cross‐presentation by dendritic cells (DCs). We have shown that DCs have the ability to store antigen for a prolonged time in endolysosomal compartments and thereby sustain MHCI antigen cross‐presentation to CD8^+^ T cells. In the current study, we investigated the role of autophagy in long‐term antigen presentation. We show that the autophagy machinery has a negative impact on storage of antigen in DCs. Atg5^–/–^DCs which are deficient in autophagy or DCs treated with common autophagy inhibitors showed enhanced antigen storage and antigen cross‐presentation. This augmented antigen cross‐presentation effect is independent of altered proteasome enzyme activity or MHCI surface expression on DCs. We visualized that the storage compartments are in close proximity to LC3 positive autophagosomes. Our results indicate that autophagosomes disrupt antigen storage in DCs and thereby regulate long‐term MHCI cross‐presentation.

## Graphical abstract

Protein antigen‐antibody complexes are efficiently taken up by dendritic cells and conserved in storage compartments for prolonged antigen cross‐presentation to CD8^+^ T cells. LC3‐positive autophagosomes can breakdown antigen storage compartments thereby regulating the cross‐presentation capacity of dendritic cells.

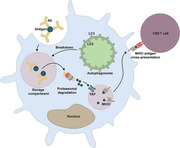

## Introduction

Dendritic cells (DCs) have been extensively investigated for their superiority in antigen cross‐presentation. Their ability to present exogenous antigen on MHCI molecules to CD8^+^ T cells has given DCs a key role in immune surveillance of cancers and infectious diseases. The mechanisms and pathways how DCs internalize, process, and present protein antigens on MHCI are studied broadly but generally under short‐term conditions, only several hours up to 18 h after antigen uptake [1, 2, 3, 4, 5]. We have previously demonstrated that DCs can store protein antigen for several days in a lysosomal‐like organelle sustaining functional antigen presentation in vitro and in vivo [6, 7]. This antigen storage organelle can be defined as an intracellular antigen‐containing compartment distinct from MHCII loading or early endosomal compartments and constitutes an internal source for the continuous supply of ligands for MHCI loading, and thereby enhancing prolonged cross‐presentation to CD8^+^ T cells. We have shown that the antigen processing from the storage organelle in DCs is TAP and proteasome dependent since inhibiting the activity of either one almost completely inhibits MHCI cross‐presentation. Therefore, it is likely that the stored antigen is translocated into the cell cytosol and degraded by the proteasome and peptidases before MHCI loading [8, 9]. DCs are known to express both constitutive and immunoproteasomes to be able to efficiently process and present antigenic peptides in MHCI molecules [10, 11].

Autophagy plays crucial role in the degradation of endogenous proteins and organelles in cells [12]. The autophagy machinery is known for its importance in regulating endogenous as well as exogenous antigen processing for MHCII presentation by fusion of autophagosomes with MHCII loading compartments (MIIC) or via LC3‐associated phagocytosis (LAP), respectively [13, 14, 15, 16, 17]. There have been reports suggesting the importance of autophagy in enhancing MHCI cross‐presentation [18, 19, 20], whereas others showed the opposite or only under specific conditions such as soluble OVA [16, 21]. Macroautophagy, which is one of the three distinct types of autophagy, is characterized by the formation of double‐membrane autophagosomes which subsequently fuse with lysosomes and release their cargo for degradation [22]. Several units, including the Atg1 complex, the PI3K complex, Atg9, the Atg2‐Atg18 complex, the Atg12 conjugation system and the Atg8 (LC3) conjugation system, are involved in the formation of autophagosomes. Atg5 gets covalently coupled to Atg12 and then forms with Atg16L1 the E3‐like ligase of the Atg8 (LC3) conjugation system. Therefore, Atg5 it is an essential protein for elongation of phagophoric membranes and substrate recruitment during autophagosome formation [23]. Atg5‐deficient DCs were impaired in their ability to present antigen on MHCII to CD4^+^ T cells due to impaired phagosomal‐to‐lysosome fusion and delivery of lysosomal proteases to the phagosomes [16].

In the current study we used DCs from mice in which Atg5 was conditionally deleted in CD11c‐positive DCs and macrophages. We show that by blocking autophagy, antigen presence in storage compartments is prolonged, as well greatly enhancing antigen cross‐presentation to CD8^+^ T cells. Using Atg8 (LC3), which gets covalently coupled to membranes of autophagosomes, we show that the storage compartments are associated with autophagosomes in DCs. Our results suggest that autophagosomes disrupt antigen storage in DCs and thereby regulate late MHCI cross‐presentation.

## Results

### Antigen storage in DCs is not disrupted by autophagy inhibitors

**Figure 1:**
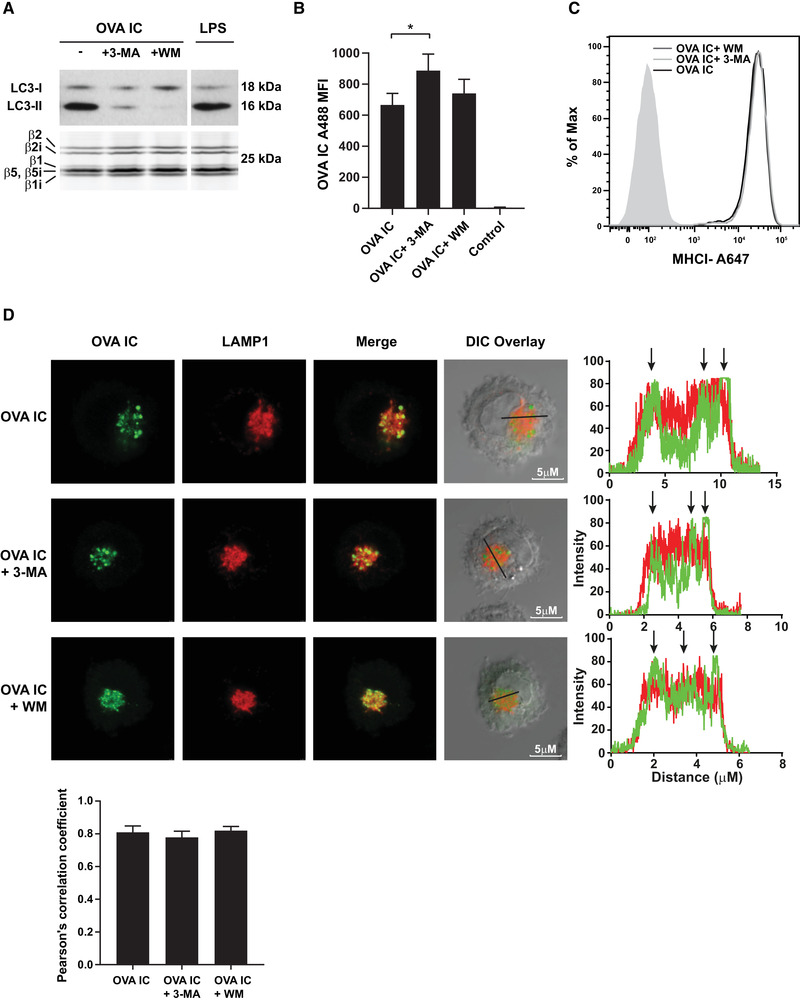
Antigen storage in DCs is not disrupted by autophagy inhibitors. DCs were pulse‐loaded with OVA IC for 2 h and chased for 48 h. Autophagy inhibitors 3‐MA or WM were added for 2 h. LPS was used as positive control for DC maturation. During the last 30 min of autophagy inhibitors incubation an activity‐based proteasome probe (BODIPY‐TMR labeled) was added to visualize the active β subunits of the proteasome. Proteins from total cell lysates (600.000 cells) were separated on SDS‐PAGE gel and BODIPY‐TMR labeled activity proteasome subunits were measured directly from the gel using a Typhoon 9410 variable mode imager. Proteins were transferred to a nitrocellulose membrane and LC3‐I and LC3‐II expression was visualized by western blot. The activity‐based generic proteasome probe was also used as an internal loading control (A). D1 cells were incubated with OVA IC (OVA Alexa Fluor 488 labeled) for 2 h and chased for 24 h. Cells were then incubated with 3‐MA or WM for 24 h. Antigen presence in DCs was measured by flow cytometry indicated by mean fluorescence intensity (MFI) with s.d. values (B). D1 cells were incubated for 2 h with OVA IC (OVA Alexa Fluor 488 labeled) and chased for 24 h followed by incubation with 3‐MA or WM for 48 h. MHCI expression levels were measured by flow cytometry. Background MFI ∼95.1, OVA IC MFI ∼2.73E4, OVA IC+ 3‐MA MFI ∼2.84E4, OVA IC+ WM MFI ∼2.75E4 (C). D1 cells were pulse‐loaded with OVA IC (Alexa Fluor 488 labeled OVA) for 2 h and chased for 24 h followed by incubation with 3‐MA or WM for 24 h. Cells were then incubated with LAMP1 (Alexa Fluor 647 labeled) antibody and imaged by confocal microscopy. Differential interference contrast (DIC) was additionally used to image cell contrast. Histograms for each fluorophore were created for a selected area (indicated by a line on the image) and overlays were made with the ImageJ software. Arrows indicate co‐localization between OVA IC (green) and LAMP1 (red). Co‐localization scores were measured by Pearson's correlation coefficient with the ImageJ software indicated by mean with s.d. values (D). Representative results are shown here from one experiment out of two independent experiments with three samples per experiment. Statistical analysis was performed using one‐way analysis of variance (ANOVA) test. Tukey's *post hoc* test was performed to correct for multiple comparisons. *p<0.05.

We have reported before that the uptake of antigen‐antibody immune complexes (OVA IC) by DCs leads to efficient antigen uptake, DC maturation, and T cell priming *in vivo* [24, 25]. Moreover, we have shown that DCs have the ability to store OVA IC for several days in endo‐lysosomal compartments [6, 7]. We suggested that antigen storage is beneficial for prolonged antigen cross‐presentation to CD8^+^ T cells *in vivo*. As MHCI‐peptide complexes have a relatively high turnover rate on the DC cell surface, these antigen storage compartments allow sustained antigen presentation for several days. We investigated whether autophagy could affect the storage of antigen in DCs by using the autophagy inhibitors Wortmannin (WM) and 3‐Methyladenine (3‐MA). DCs were first pulse‐loaded with OVA IC for 2 h and chased for 24 h (in order to let antigen end up in endo‐lysosomal compartments), followed by incubation of autophagy inhibitors. DCs that were incubated with 3‐MA or WM showed decreased LC3 coupling to autophagic membranes, as measured by LC3‐II expression, indicating reduced formation of autophagosomes (Fig. 1A, upper panel). Proteasome activity, as detected with an activity‐based fluorescent probe showing the active subunits of the constitutive proteasome (β1, β2, β5) and immunoproteasome (β1i, β2i, β5i), was not affected by the inhibition of autophagy (Fig. 1A, lower panel). The amount of antigen conserved in DCs in the presence of 3‐MA is significantly higher than without inhibitor (Fig. 1B). In the presence of WM, the amount of antigen is slightly increased, albeit not significant (Fig. 1B). Inhibiting autophagy did not affect the expression levels of MHCI on DCs (Fig. 1C). After 2 h pulse and 24 h chase, antigen was stored in DCs in LAMP1 positive compartments, located perinuclear in the cell cytosol (Fig. 1D, upper panel). It showed similar outcome when DCs were incubated with either 3‐MA or WM (Fig. 1D, middle and lower panel). High co‐localization scores were measured by Pearson's correlation coefficient (∼0.8) with no significant differences between the conditions. The storage compartments are distinct from EEA‐1 loading compartments, no co‐localization was found with antigen and EEA‐1 marker whether DCs were incubated with or without autophagy inhibitors, also indicated by low co‐localization scores (∼0.2) (Supplemental Fig. 1). These results indicate that using inhibitors to block autophagosome formation does not detectably disrupt antigen storage in LAMP1 positive endosomal compartments in DCs.

### Long‐term cross‐presentation is enhanced by autophagy inhibitors

**Figure 2:**
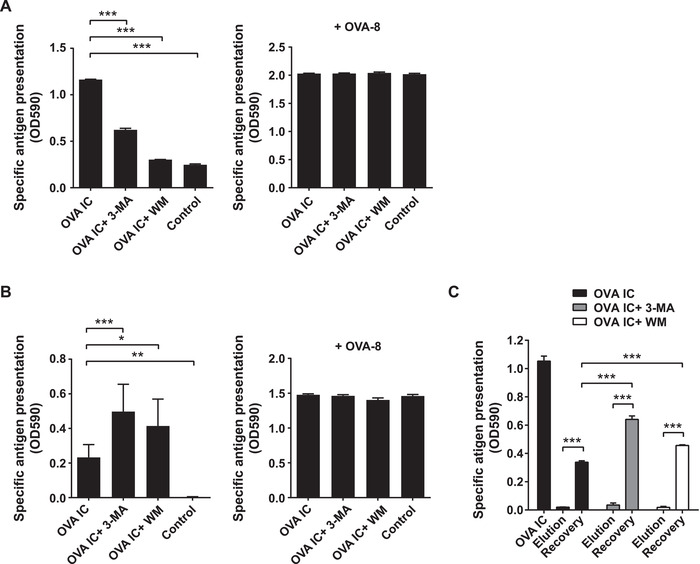
DC cross‐presentation is enhanced by autophagy inhibitors. DCs (n = 3) were pre‐incubated with 3‐MA or WM for 2 h before incubation with OVA IC for 2 h. Cells were washed and antigen presentation was measured with absorbance spectrophotometer by adding B3Z CD8^+^ T cell hybridoma overnight (indicated by OD590, left graph). Minimal peptide OVA‐8 SIINFEKL (right graph) was added as positive control. The mean with s.d. values are shown (A). DCs (n = 3) were incubated with OVA IC for 2 h and chased for 24 h. Cells were then incubated with 3‐MA or WM for 24 h. Cells were washed and antigen presentation was measured by absorbance spectrophotometer with B3Z T cells (indicated by OD590) and the mean with s.d. values are shown (B). DCs (n = 3) were pulse loaded with OVA IC for 2 h followed by 24 h chase. Cells were then incubated with 3‐MA or WM for 24 h. Cell surface peptides on MHCI molecules were stripped by mild acid citrate/phosphate elution buffer and incubated again with 3‐MA or WM during 6 h of peptide recovery. Afterwards, the cells were washed and antigen presentation was measuredby absorbance spectrophotometer overnight with B3Z T cells (indicated by OD590) and the mean with s.d. values are shown (C). Representative results from one experiment with three technical replicates are shown here out of three independent experiments. Statistical analysis was performed using one‐way analysis of variance (ANOVA) test. Tukey's *post hoc* test was performed to correct for multiple comparisons. *p<0.05, **p<0.01, ***p<0.001.

Several reports have shown that blocking autophagy results in reduced MHCI cross‐presentation by DCs, however this was generally analyzed a few hours after antigen uptake [19, 20]. We could show comparable results when DCs were pre‐incubated with 3‐MA or WM and incubated with OVA IC for 2 h (Fig. 2A, left graph). In the presence of autophagy inhibitors, early antigen cross‐presentation by DCs was significantly impaired. DC cell surface loading with minimal peptide OVA‐8 (SIINFEKL) indicated that the treated DCs were not hampered in their antigen presentation capacity (Fig. 2A, right graph). To investigate the impact of autophagy on long term DC cross‐presentation, DCs were pulse‐loaded with OVA IC for 2 h and chased for 24 h, followed by incubation with autophagy inhibitors. In contrast, antigen presentation was significantly enhanced in the presence of 3‐MA or WM (Fig. 2B, left graph), whereas exogenous minimal peptide OVA‐8 loading showed again no differences (Fig. 2B, right graph). To establish that presented antigenic peptides were derived from internal sources we stripped the cell surface of DC cells which were pulse‐loaded with OVA IC and chased for 24 h in the presence or absence of autophagy inhibitors. Cell surface peptides were stripped with a mild acidic elution buffer and the recovery of newly synthesized peptides from the storage compartment loaded on MHCI was measured. All conditions showed undetectable antigen presentation when the DC cell surface was stripped with elution buffer (Fig. 2C). DCs without inhibitors were able to recover newly synthesized peptides on the surface, although to a lesser extent (∼30%) compared to before the elution (Fig. 2C, dark bars) as we have published previously [6]. Both 3‐MA and WM significantly enhanced peptide recovery from the storage compartment compared to recovery without inhibitors (Fig. 2C, grey and white bars). These results indicate that blocking autophagy at a later time point after antigen uptake results in enhanced antigen cross‐presentation from internal storage compartments. This is in contrast with the decrease in antigen cross‐presentation observed at earlier time points in the presence of autophagy inhibitors.

### Antigen storage is prolonged in autophagy‐deficient DCs

**Figure 3:**
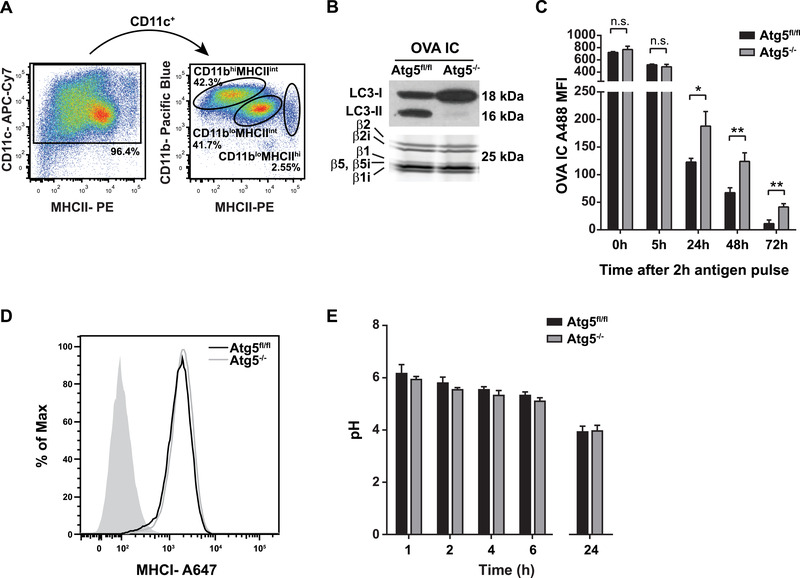
Antigen storage is enhanced in autophagy‐deficient DCs. Bone marrow dendritic cells (BMDCs) were gated according to the following markers: CD11c^+^ CD11b^lo^ MHCII^int^ by flow cytometry (A). BMDCs were sorted and pulsed with OVA IC for 2 h and chased for 48 h followed by 30 min incubation with activity‐based proteasome probe (BODIPY‐TMR labeled). Proteins from total cell lysates (600.000 cells) were separated on SDS‐PAGE gel and BODIPY‐TMR labeled activity proteasome subunits were measured directly from the gel using a Typhoon 9410 variable mode imager. Proteins were transferred to a nitrocellulose membrane and LC3‐I and LC3‐II expression was visualized by western blot.The activity‐based generic proteasome probe was also used as an internal loading control (B). BMDCs from three Atg5^–/–^ or three Atg5^fl/fl^ mice were incubated with OVA IC (OVA Alexa Fluor 488 labeled) for 2 h and chased for 0, 5, 24, 48 or 72 h. Antigen presence in DCs was measured by flow cytometry (indicated by MFI) and the mean with s.d. values are shown (C). BMDCs from two Atg5^–/–^ or two Atg5^fl/fl^ mice were incubated for 2 h with OVA IC (OVA Alexa Fluor 488 labeled) and chased for 24 h. MHCI expression levels were measured by flow cytometry. Background MFI ∼103, Atg5^fl/fl^ MFI ∼1720, Atg5^–/–^ MFI ∼1918 (D). pH of antigen containing compartments in BMDCs from one Atg5^–/–^ or one Atg5^fl/fl^ mouse was measured. Cells were incubated with IC (partial OVA FITC (pH sensitive) and Alexa Fluor 647 labeled as described in material and methods) for 1 h and 1, 2, 4, 6, or 24 h chase. The uptake of OVA antigen labeled with FITC and Alexa Fluor 647 was measured by flow cytometry. The ratio between OVA FITC and OVA Alexa Fluor 647 was calculated to determine the pH value of antigen compartments. The mean with s.d. values are shown (E). Representative results from one experiment with three technical replicates are shown here out of two independent experiments. Statistical analysis was performed using one‐way analysis of variance (ANOVA) test. Tukey's *post hoc* test was performed to correct for multiple comparisons. n.s non‐significant, *p<0.05, **p<0.01.

We generated bone marrow dendritic cells (BMDCs) from autophagy‐deficient mice lacking Atg5 in CD11c positive cells (Atg5^–/–^) to further investigate the effect of autophagy on antigen storage in DCs. Atg5 forms a complex together with Atg12 and Atg16L1 which is required for the association of LC3 with the autophagosomal membrane during the early stages of autophagosome formation. BMDCs are known to be a heterogeneous population [26], we used the following gating strategy to isolate DCs: CD11c^+^ CD11b^lo^ MHCII^int^ (Fig. 3A, Supplemental Fig. 2). Atg5^–/–^ DCs have higher LC3‐I and totally lacked LC3‐II expression compared to control mice (Atg5^fl/fl^), whereas proteasome activity remained the same (Fig. 3B). Atg5^–/–^ DCs were pulsed for 2 h with OVA IC and chased for different time points. After the initial 2 h pulse of antigen and 5 h chase, the amount of antigen uptake is similar in both Atg5^–/–^ and Atg5^fl/fl^ DCs (Fig. 3C). However, after 24 h or longer, significantly more antigen was detectable in DCs lacking Atg5. MHCI expression was comparable in Atg5^–/–^ and Atg5^fl/fl^ after 24 h antigen chase (Fig. 3D). The pH of the antigen containing compartments in both Atg5^–/–^ and Atg5^fl/fl^ were similar after 1 h antigen pulse, and decreased gradually but remained comparable in time towards pH 4 in the storage compartments (Fig. 3E). These results indicate that in absence of autophagy mediator Atg5 the presence of antigen in DCs is prolonged, which is not related to significant changes in phagosomal acidification.

### Long‐term MHCI antigen cross‐presentation is enhanced in autophagy‐deficient DCs

**Figure 4:**
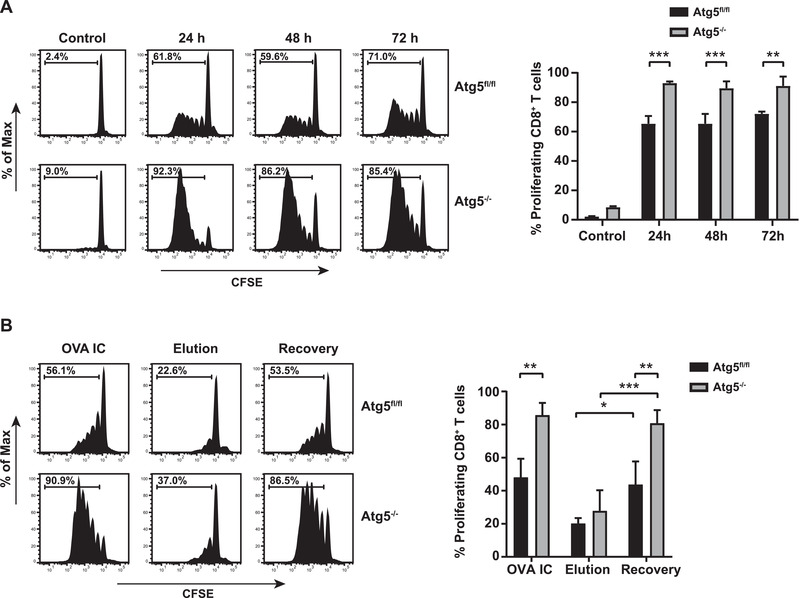
MHCI antigen cross‐presentation is enhanced in autophagy‐deficient DCs. BMDCs from two Atg5^–/–^ or two Atg5^fl/fl^ mice were sorted and pulsed for 2 h with OVA IC and chased for 24, 48 or 72 h. CFSE labeled CD8^+^ T cells from OTI mice were added and T cell proliferation was measured after 3 days by flow cytometry. Representative proliferations histograms (left) and mean with s.d. values (right) are shown (A). BMDCs from two Atg5^–/–^ or two Atg5^fl/fl^ mice were pulse‐loaded with OVA IC for 2 h followed by 48 h chase. Cells were then stripped for surface MHCI molecules with mild acid citrate/phosphate elution buffer and recovered for 6 h. CFSE labeled CD8^+^ T cells from OTI mice were added and T cell proliferation was measured after 3 days by flow cytometry. Representative proliferations histograms (left) and mean with s.d. values (right) are shown (B). Representative results are shown here from one experiment with two technical replicates are shown here out of two independent experiments. Statistical analysis was performed using one‐way analysis of variance (ANOVA) test. Tukey's *post hoc* test was performed to correct for multiple comparisons. *p<0.05, **p<0.01, ***p<0.001.

Since blocking autophagy reduced antigen degradation in the storage compartments, we investigated the effect on antigen cross‐presentation to CD8^+^ T cells. Atg5^–/–^ DCs were pulse loaded with OVA IC and chased for 24, 48 or 72 h. Antigen cross‐presentation after 24 h was detectably higher by Atg5^–/–^ DCs compared to Atg5^fl/fl^ DCs (Fig. 4A). Antigen presentation capacity of the Atg5^–/–^ DCs sustained in time, even 72 h after the initial antigen pulse CD8^+^ T cell proliferation remained superior to Atg5^fl/fl^ DCs. Comparable results were found when using soluble OVA protein, Atg5^–/–^ DCs induced higher CD8^+^ T cell proliferation compared to Atg5^fl/fl^ DCs (Supplemental Fig. 3). Moreover, mild acid elution was carried out to show peptide recovery from internal antigen sources. Atg5^–/–^ DCs were pulse loaded with OVA IC, chased for 48 h and stripped from their cell surface peptides with an elution buffer. Prior to peptide elution, Atg5^–/–^ DCs again showed higher antigen cross‐presentation to CD8^+^ T cells compared to Atg5^fl/fl^ DCs (Fig. 4B, left panel). After peptide elution, antigen presentation reduced to similarly low levels in both Atg5^–/–^ and Atg5^fl/fl^ DCs (Fig. 4B, middle panel). Six hours recovery was sufficient to restore the initial antigen presentation capacity of the DCs, however, Atg5^–/–^ DCs induced much higher CD8^+^ T cell proliferation compared to Atg5^fl/fl^ DCs (Fig. 4B, right panel). These data indicate that more antigen from internal sources remains available for antigen cross‐presentation by autophagy‐deficient DCs.

### Antigen storage compartments are in close proximity to autophagosomes

Our results indicate that autophagosomes have negative effects on antigen storage in DCs and thereby affecting antigen cross‐presentation. It is therefore likely that the antigen containing compartments and autophagosomes are in close proximity in the cell. DCs were pulse‐ loaded with OVA IC (Alexa Fluor 488 labeled) and chased in time up to 24 h. To increase the visualization of LC3‐positive autophagosomes the DCs were additionally treated with Chloroquine to inhibit autophagic flux by decreasing autophagosome‐lysosome fusion [27]. Using an LC3 marker that stains both LC3‐I and LC3‐II (Alexa Fluor 647 labeled), autophagosomes could be detected in DCs. After 30 min antigen pulse, LC3 showed punctuated “hotspots” spread in the cell cytosol distinct from antigen (co‐localization score of ∼0.4) (Fig. 5A, upper panel). After 1 h, LC3 started to cluster more around the antigen compartments (co‐localization score ∼0.4), and after 3 h, the majority of LC3 was located on the same perinuclear location as the antigen containing compartments (co‐localization score ∼0.6) (Fig. 5A, 2nd and 3rd panel). After 24 h, most LC3 is co‐localizing or in close proximity with the storage compartments (co‐localization score ∼0.9) (Fig. 5A, bottom panel). Similar experiment with 30 min antigen pulse in Atg5^–/–^ DCs showed no punctuated LC3 “hotspots” (LC3‐II), but instead a diffuse cytosolic staining (LC3‐I) (Fig. 5B, 2nd panel) compared to Atg5^fl/fl^ DCs (Fig. 5B, upper panel). After 24 h LC3 “hotspots” were co‐localizing with OVA IC in Atg5^fl/fl^ DCs (co‐localization score ∼0.8) but not in Atg5^–/–^ DCs (co‐localization score ∼0.4) (Fig. 5B, third and lower panel). These results show that the antigen containing compartments are co‐localizing with or in close proximity to autophagosomes.

Figure 5Antigen storage compartments are in close proximity with autophagosomes. Dendritic cells were pulse‐loaded with OVA IC (Alexa Fluor 488 labeled OVA) for 30 min and chased for 1, 3, or 24 h. In addition, 10 μM Chloroquine was added for 30 min to induce higher LC3 expression levels and cells were stained with LC3 marker (Alexa Fluor 647 labeled) (A). BMDCs from two Atg5^–/–^ or two Atg5^fl/fl^ mice were incubated with OVA IC (Alexa Fluor 488 labeled OVA) for 30 min or pulse‐loaded for 2 h and chased for 24 h. In addition, 10 μM Chloroquine was added for 30 min to induce higher LC3 expression levels and cells were stained with LC3 marker (Alexa Fluor 647 labeled) (B). Cells were imaged by confocal microscopy and differential interference contrast (DIC) was additionally used to image cell contrast. Histograms for each fluorophore were created for a selected area (indicated by a line on the image) and overlays were made with the ImageJ software. Arrows indicate co‐localization between OVA IC (green) and LC3 (red). Co‐localization scores were measured by Pearson's correlation coefficient with the ImageJ software indicated by mean with s.d. values. Representative results are shown here from one experiment with two replicates out of two independent experiments.

**Figure:**
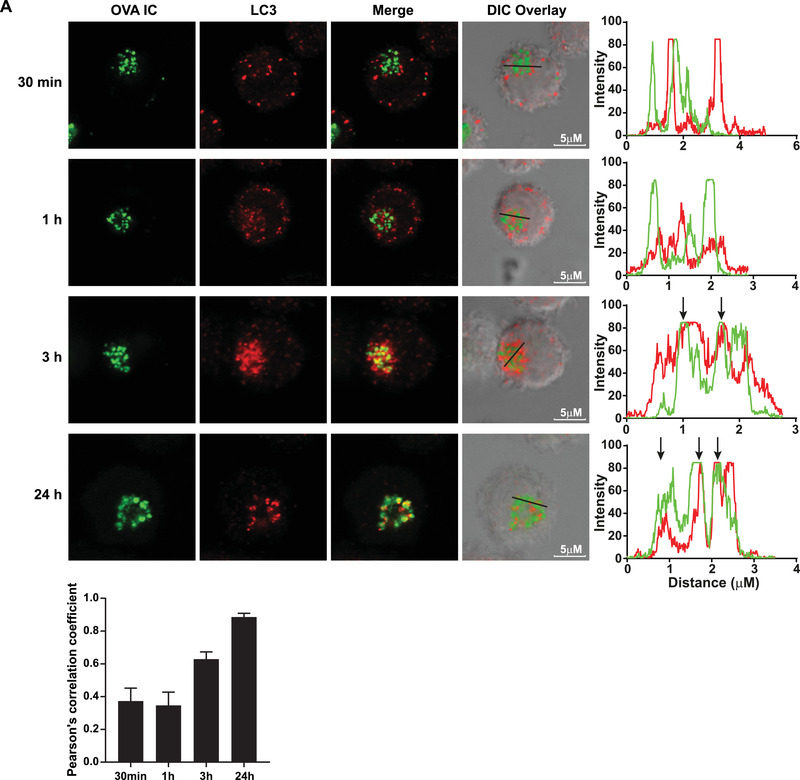

**Figure:**
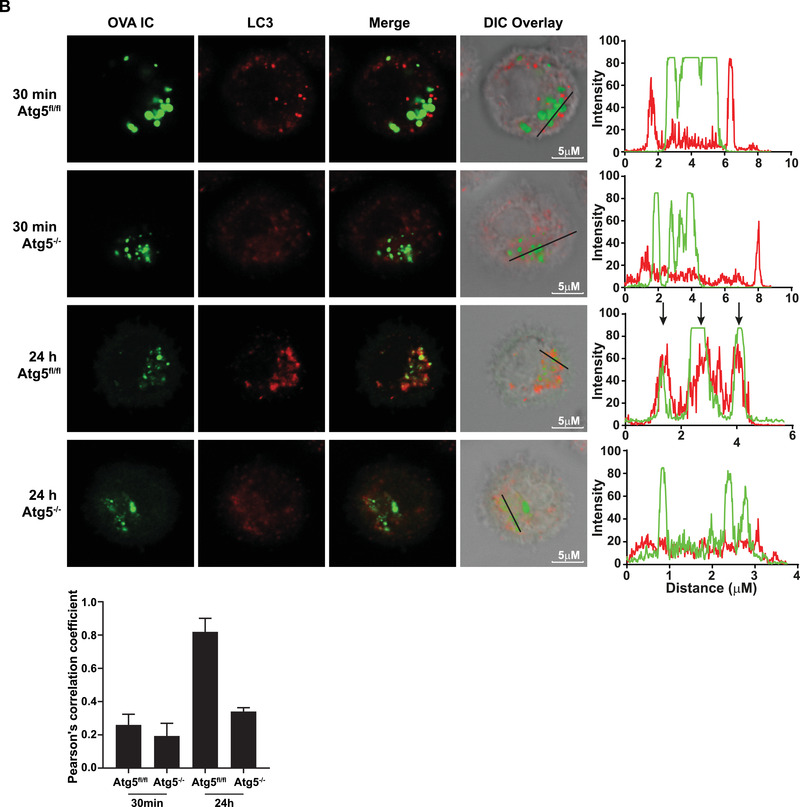

## Discussion

Dendritic cells (DCs) have been recognized for their superiority in cross‐presenting exogenous antigen to CD8^+^ T cells. We have previously reported that DCs can store antigen targeted to Fcγ receptors, C‐type lectin receptors, or Toll‐like receptors for several days in a lysosome‐like storage compartment which contributes to sustained antigen presentation to T cells [6, 7, 28]. Intact antigen can still be detected in DCs up to 7 days after initial pulse *in vitro* [6]. Antigen taken up by DCs *in vivo* after injecting mice with anti‐OVA antibody and subsequently with OVA protein showed the ability to induce functional CD8^+^ T cells even 1 week after injection [7]. Since MHCI molecules on DCs have a high turnover rate and the migration time of DCs from infection site to lymph nodes where they interact with T cells might take up to several days, the continuous supply of newly processed antigen from storage compartments could be beneficial to induce effective and prolonged T cell responses. In the current study we demonstrated, with the use of antibody‐bound antigen (OVA IC) which is effectively engulfed by Fc receptor‐mediated uptake, that autophagy has a severe impact on the amount of antigen stored in the storage compartments and thereby affecting antigen cross‐presentation outcome.

Since autophagosomes and MHCI expression was already highly present in DCs targeted by OVA IC, our initial attempts failed to further enhance autophagy with compounds such as Rapamycin, Torin 1, and Torin 2 (data not shown). In these conditions, it was difficult to interpret antigen cross‐presentation due to pleiotropic effects of the autophagy inducers. Therefore, we focused on studying the effects of autophagy on DC cross‐presentation by using autophagy inhibitors and ultimately Atg5^–/–^ mice. DCs that were treated with common autophagy inhibitors or DCs derived from Atg5^–/–^ mice showed prolonged antigen storage and significantly enhanced antigen cross‐presentation to CD8^+^ T cells. This was rather unexpected since it was reported that autophagy inhibition can negatively influence MHCI cross‐presentation [18, 19, 20]. We could confirm these findings but only in short term antigen presentation assays. The novelty of our observations is the opposite enhancing effect on long‐term antigen cross‐presentation by blocking autophagy in DC which can be explained by inhibition of autophagosomal degradation of internal antigen containing compartments.

An alternative explanation for enhanced MHCI antigen cross‐presentation upon autophagy inhibition was described by Loi et al. showing elevated MHCI surface levels on Atg5 or Atg7 deficient DCs [29]. This might be a cellular mechanism to balance the MHCI and MHCII antigen presentation by DCs, and was mainly observed on DCs and alveolar macrophages *ex vivo*. However, in our study conditions we did not find significant differences in MHCI surface expression levels on Atg5 deficient DCs upon maturation, suggesting the enhanced cross‐presentation is rather caused by increased peptide production. Moreover, we showed that proteasomal enzyme activity was not affected in autophagy deficient DCs or by treating DCs with autophagy inhibitors, thereby excluding a significant role of proteasome activity in enhancing cross‐presentation upon autophagy inhibition.

We showed in the current study that antigen containing storage compartments are co‐localizing or at least in very close proximity with LC3‐positive autophagosomes. However, it cannot be ruled out that LC3 positive compartments are vesicles mediated by LC3‐associated phagocytosis (LAP), an autophagy pathway initiated by pattern recognition receptors and mainly described as machinery for extracellular MHCII presentation [17, 30]. Therefore, additional staining for p62, which is present on autophagosomes, could distinguish LAP from autophagosomes. It has been shown that LAP formation is dependent on the recruitment of NOX2 to the vesicles and the generation of ROS [17, 31, 32]. However, preliminary data with NOX2‐deficient mice did not show any effect on sustained antigen cross‐presentation by DCs (data not shown), therefore it is unlikely that LAP is playing a crucial role in our setting.

We did not find any detectable differences in antigen containing compartment‐pH between wildtype and Atg5 deficient DCs. It seems that antigen degradation in the storage compartments is not controlled by lysosomal enzyme activity within the compartments, but rather degraded by autophagosomes in a different manner. One possible explanation is that autophagosomes prevent antigen translocation from the endosomal compartments to the cytosol for further processing. It has been reported that during autophagy it is important that the edges of the isolation membrane of autophagosomes are sealed. This is to prevent leakage of hydrolases that can cause cellular damage and apoptosis [33, 34]. Another possibility is that, under normal conditions, the storage compartments are slowly leaking antigen to the cytosol for antigen processing and MHCI presentation. However this process might be disrupted by autophagy since it has been reported that autophagosomes can degrade leaky endosomes [35]. This might regulate the long‐term storage of antigen in the storage compartments and thereby the sustained cross‐presentation ability of DCs. How protein antigen is stored in the storage compartments is still not clear. There might be specific hydrolases or cathepsins lacking in the storage compartments.

Several studies have investigated the role of autophagy in DC cross‐presentation with contradictory results. Some groups showed elevated CD8^+^ T cell responses upon autophagy inhibition in DCs with different antigen targeting systems [29], while others showed autophagy‐independent cross‐presentation [16] or even lowered immune responses upon blocking autophagy [19, 21]. It seems that the outcome depends on the type of antigen, cell subset and time point of measuring antigen presentation. Most studies show DC antigen presentation already after a few hours, whereas we have studied prolonged antigen presentation capacity of DCs several days after the initial antigen pulse. In the current report we showed that blocking autophagy inhibits breakdown of antigen containing compartments and thereby enhancing the presence of protein antigen in DCs available for antigen cross‐presentation to CD8^+^ T cells. We therefore propose that autophagy can regulate long term antigen cross‐presentation capacity of DCs.

## Materials and methods

### Cells

The D1 cells, a long‐term growth factor dependent immature splenic DC line derived from C57BL/6 (BL/6) mice, were kindly provided by P. Ricciardi‐Castagnoli (University of Milano‐Bicocca, Italy) and cultured as described [36]. Bone marrow cells from Atg5^–/–^ and Atg5^fl/fl^ mice (provided by C. Münz and bred and housed in the University of Zurich animal facility according to Swiss animal laws and institutional guidelines) were cultured in the presence of 30% R1 supernatant from NIH3T3 fibroblasts transfected with GM‐CSF for 10 days. Atg5^–/–^ mice were conditionally deleted in CD11c‐positive DCs and macrophages by crossing CD11c‐cre mice with Atg5^fl/fl^ mice. From this source, bone marrow‐derived dendritic cells (BMDCs) were generated and gated with flow cytometry according to the following markers: CD11c^+^ CD11b^lo^ MHCII^int^. The guidelines of Cossariza *et al*. were followed for the use of flow cytometry [37]. CD8^+^ T cells (CD8^+^/ CD45.1^+^) were purified (Mouse CD8 T Lymphocyte Enrichment Set, BD Biosciences) from the spleen of OTI mice (CD8^+^ T cell transgenic mice expressing a TCR recognizing the OVA derived K^b^ associated epitope SIINFEKL) that were bred and kept at the LUMC animal facility under SPF conditions. B3Z is a CD8^+^ T cell hybridoma specific for SIINFEKL on H2‐K^b^ MHCI molecules and expresses LacZ upon activation.

### Ag‐IgG immune complexes

OVA‐IgG immune complexes (OVA IC) were formed by incubating 1 μg/ml OVA (unconjugated; Worthington Biochemical, or Alex Fluor 488 conjugated; Life Technologies) and 300 μg/ml anti‐OVA IgG (rabbit polyclonal, LSBio) for 30 min at 37°C *in vitro*. DCs were loaded with OVA IC as indicated in each experiment.

### LC3 expression in DCs

Immature D1 DCs were pulsed with OVA IC (unconjugated OVA) for 2 h, washed and chased for 48 h followed by 2 h incubation with either 5 mM 3‐Methyladenine (3‐MA, Calbiochem), 0.5 μM Wortmannin (WM, Invivogen) or medium control. As a positive control for DC maturation, 5 μg/ml LPS (Sigma‐Aldrich) was used. During the last 30 min of autophagy inhibitors incubation 2 μM activity‐based proteasome probe MVB003 (BODIPY‐TMR labeled), which was kindly provided by Hermen Overkleeft (Leiden Institute of Chemistry, The Netherlands), was added [38]. BMDCs from Atg5^–/–^ or Atg5^fl/fl^ mice were generated, gated in flow cytometry as described above and sorted by BD FACSAria II SORP (BD Biosciences). BMDCs were pulsed with OVA IC (unconjugated OVA) for 2 h and chased for 48 h followed by 30 min incubation with the activity‐based proteasome probe. Proteins from total cell lysates from D1 DCs or BMDCs were separated by 15% SDS‐PAGE gel and BODIPY‐TMR labeled activity proteasome subunits were measured directly from the gel by using a Typhoon 9410 variable mode imager (GE Healthcare Bio‐Sciences). Proteins were then transferred to a nitrocellulose membrane and incubated with polyclonal rabbit IgG anti‐LC3 (MBL) followed by Peroxidase‐conjugated Goat anti‐Rabbit IgG (H+L, Jackson ImmunoResearch) and visualized with an enhanced chemiluminescent substrate for the detection of HRP (ThermoFisher Scientific) in western blot.

### Antigen presence in dendritic cells

Immature D1 DCs were pulse‐loaded with OVA IC (OVA Alexa Fluor 488 labeled) for 2 h and chased for 24 h. Cells were then incubated with 5 mM 3‐MA, 0.5 μM WM, or medium control for 24 h. BMDCs from Atg5^–/–^ or Atg5^fl/fl^ mice were incubated with OVA IC (OVA Alexa Fluor 488 labeled) for 2 h and chased for 0, 5, 24, 48 or 72 h. Antigen presence in DCs was measured by flow cytometry.

### DC antigen presentation

For early antigen presentation, immature D1 DCs were pre‐incubated with 5 mM 3‐MA or 0.5 μM WM for 2 h before incubation with OVA IC (unconjugated) for 2 h. Antigen presentation was measured by adding B3Z CD8^+^ T cell hybridoma overnight. Minimal peptide OVA‐8 SIINFEKL (2 ng/ml), which binds directly to cell surface MHCI, was added as positive control. For late antigen presentation, immature D1 DCs were incubated with OVA IC (unconjugated OVA) for 2 h and chased for 24 h. Cells were then incubated with 5 mM 3‐MA, 0.5 μM WM, or medium control for 24 h. Antigen presentation was measured by adding B3Z CD8^+^ T cell hybridoma overnight. Minimal peptide OVA‐8 SIINFEKL was added as positive control. Optical density was measured with absorbance spectrophotometer at 590nm. BMDCs from Atg5^–/–^ or Atg5^fl/fl^ mice were sorted as described above and pulsed for 2 h with OVA IC (unconjugated OVA) and chased for 24, 48 or 72 h, or cells were pulsed for 2h with 500μg/ml OVA (unconjugated) and chased for 48 h. CFSE labeled CD8^+^ T cells (CD8^+^/ CD45.1^+^) from OTI mice were added and T cell proliferation was measured 3 days later by flow cytometry.

### DC peptide elution assay

Immature D1 DCs were pulse‐loaded with OVA IC (unconjugated OVA) for 2 h followed by 24 h chase. Cells were then incubated with 5 mM 3‐MA, 0.5 μM WM or medium control for 24 h. Cell surface peptides on MHCI molecules were stripped by mild acid citrate/phosphate buffer with pH3.3. Cells were again incubated with 3‐MA, WM or medium control during 6 h of peptide recovery and fixated in 0.2% paraformaldehyde before T cell activation read‐out with B3Z T cells. Optical density was measured with absorbance spectrophotometer at 590nm. BMDCs from Atg5^–/–^ or Atg5^fl/fl^ mice were sorted as described above and pulse loaded with OVA IC (unconjugated OVA) for 2 h followed by 48 h chase, stripped for surface MHCI molecules and recovered for 6 h.

### MHCI expression on DCs

Immature D1 DCs were incubated with OVA IC (Alexa Fluor 488 labeled OVA) for 24 h followed by 48 h with 5 mM 3‐MA, 0.5 μM WM or medium control. BMDCs from Atg5^–/–^ or Atg5^fl/fl^ mice were gated and sorted as described above and treated similar as the D1 DCs. Cells were harvested and incubated with primary monoclonal MHCI H‐2K^b^ (B8.24.3) antibody and secondary Goat anti‐Mouse IgG Alexa Fluor 647 conjugated antibody (ThermoFisher Scientific). MHCI expression was measured by flow cytometry.

### Antigen co‐localization with markers in the presence of autophagy inhibitors

D1 cells were pulse‐loaded with OVA IC (Alexa Fluor 488 labeled OVA) for 2 h and chased for 24 h followed by incubation with 5 mM 3‐MA or 0.5 μM WM for 24 h. Cells were transferred to glass bottom dishes (MatTek corporation, Ashland, USA) and incubated with one of the following primary antibodies as indicated in each experiment: LAMP1 (CD107a, Alexa Fluor 647, Biolegend), EEA‐1 (C‐15, Santa Cruz), and secondary antibody: anti‐goat IgG (Alexa Fluor 647, Invitrogen). Cells were imaged using Leica SP5 STED confocal microscope with a 63x objective lens. Differential interference contrast (DIC) was additionally used to image cell contrast. Images were acquired in 10x magnification and processed with Leica LAS AF Lite software. Co‐localization quantification was performed using Pearson's correlation coefficient with JACoP in ImageJ [39].

### Antigen co‐localization with LC3 positive compartments

D1 cells were pulse‐loaded with OVA IC (Alexa Fluor 488 labeled OVA) for 30 min and chased for 1, 3, or 24 h. BMDCs from Atg5^–/–^ or Atg5^fl/fl^ mice were incubated with OVA IC (Alexa Fluor 488 labeled OVA) for 30 min and chased for 24 h. In addition, 10 μM Chloroquine was added for 30 min to block autophagosome turnover and LC3 degradation. Cells were then stained with LC3 antibody (4E12, MBL) and secondary antibody anti‐mouse IgG (Alexa Fluor 647, Invitrogen). Cells were imaged using Leica SP5 STED confocal microscope with a 63x objective lens. Differential interference contrast (DIC) was additionally used to image cell contrast. Images were acquired in 10x magnification and processed with Leica LAS AF Lite software. Co‐localization quantification was performed using Pearson's correlation coefficient with JACoP in ImageJ [39].

### pH measurements in storage compartments

BMDCs from Atg5^–/–^ or Atg5^fl/fl^ mice were gated and sorted as described above and incubated with IC (1 h pulse and 1, 2, 4, 6, 8, or 24 h chase) formed from: 3.8 μg/ml OVA FITC (ThermoFisher Scientific), 0.2 μg/ml OVA Alexa Fluor 647 (Biolegend) and 1.6 mg/ml anti‐OVA IgG (rabbit polyclonal, LSBio). The uptake of OVA antigen labeled with FITC and Alexa Fluor 647 were measured by flow cytometry indicated by MFI. Since the FITC signal will be reduced upon encountering acidic environments, the MFI ratio between OVA FITC and OVA Alexa Fluor 647 was calculated to determine the pH value of storage compartments. A standard curve of MFI ratio‐pH was made by incubating OVA IC positive‐BMDCs with a range of different pH buffers.

### Statistical analysis

Statistical analysis was performed using one‐way analysis of variance (ANOVA) test. Tukey's *post hoc* test was performed to correct for multiple comparisons. The following indications are used in all figures: n.s: non‐significant, *p<0.05, **p<0.01, ***p<0.001.

## Conflict of interest

The authors declare no financial or commercial conflict of interest.

### Peer review

The peer review history for this article is available at https://publons.com/publon/10.1002/eji.202048961.

**Abbreviations**
3‐MA: 3‐methyladenine
BMDCs: bone marrow dendritic cells
DIC: differential interference contrast
LAP: LC3‐associated phagocytosis
WM: Wortmannin

## Supporting information

Supporting information

## Data Availability

Data available on request from the authors
