# Supplementary material for: Autophagy regulates long‐term cross‐presentation by murine dendritic cells
Source: Eur J Immunol. 2021 Feb 10;51(4):835–47. doi: 10.1002/eji.202048961 (PMC8248248; doi:10.1002/eji.202048961)
Supplement: Supplementary file 1 — Supporting information [file EJI-51-835-s001.pdf]

Supplemental Figure 1

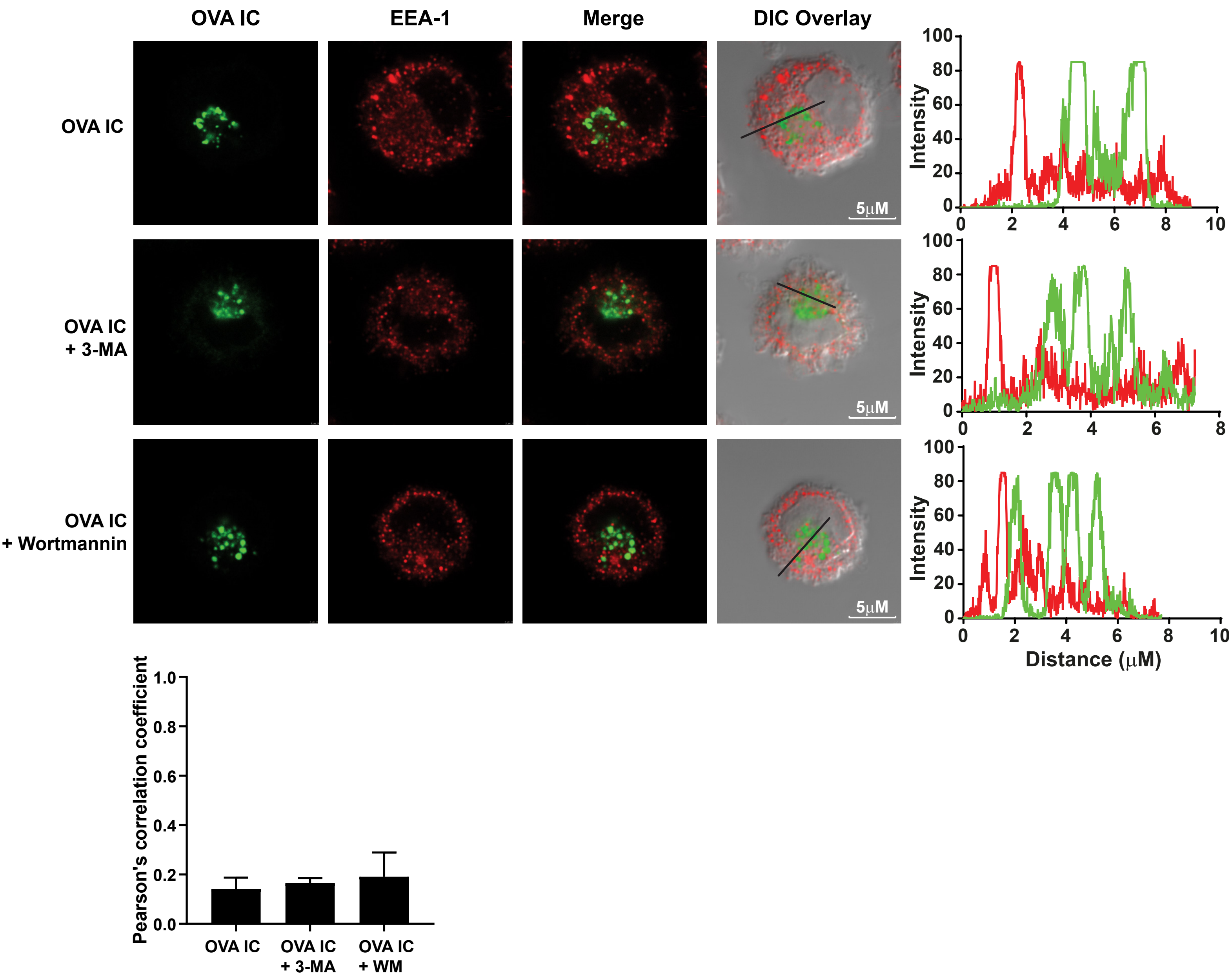

Supplemental Figure 1. Antigen storage compartment co-staining with EEA-1. DCs were pulse-loaded with OVA IC (Alexa Fluor 488 labeled OVA) for 2 h and chased for 24 h followed by incubation with 3-MA or WM for 24 h. Cells were then incubated with EEA-1 antibody and imaged by confocal microscopy. Differential interference contrast (DIC) was additionally used to image cell contrast. Histograms for each fluorophore were created for a selected area (indicated by a line on the image) and overlays were made with the ImageJ software between OVA IC (green) and EEA-1 (red). Co-localization scores were measured by Pearson's correlation coefficient with the ImageJ software indicated by mean with s.d. values. Representative results are shown here from one experiment out of two independent experiments.

Supplemental Figure 2

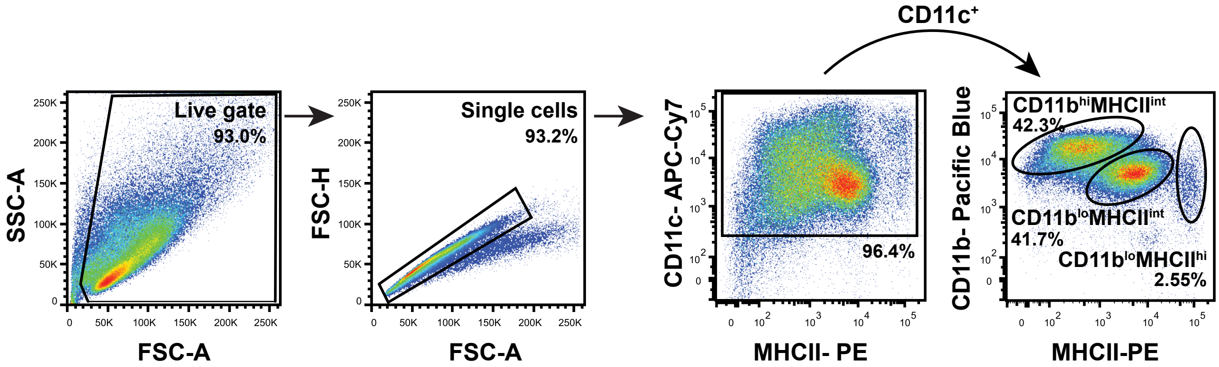

Supplemental Figure 2. BMDCs gating strategy. BMDCs were generated and gated with flow cytometry according to the following markers: $CD11c^{+}$ $CD11b^{lo}$ $MHCII^{int}$.

Supplemental Figure 3

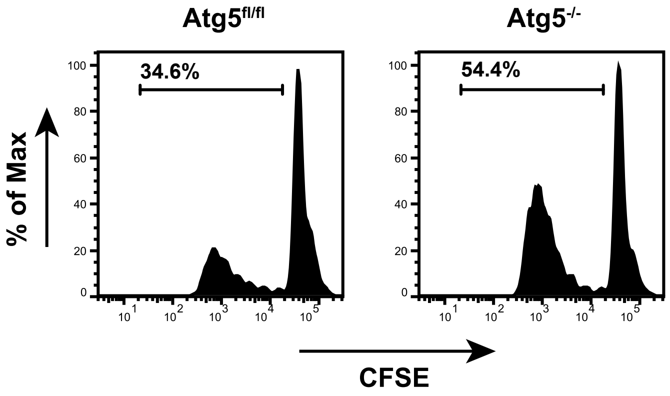

Supplemental Figure 3. Antigen presentation with soluble antigen is enhanced in autophagy-deficient DCs. BMDCs from $Atg5^{-/-}$ or $Atg5^{fl/fl}$ mice were pulse-loaded with 500μg/ml OVA for 2 h followed by 48 h chase. CFSE labeled $CD8^+$ T cells from OTI mice were added and T cell proliferation was measured after 3 days by flow cytometry.
